# ANXC7 Is a Mitochondrion-Localized Annexin Involved in Controlling Conidium Development and Oxidative Resistance in the Thermophilic Fungus *Thermomyces lanuginosus*

**DOI:** 10.3389/fmicb.2018.01770

**Published:** 2018-09-11

**Authors:** Xiang-Li Xie, Huan Yang, Li-Na Chen, Yi Wei, Shi-Hong Zhang

**Affiliations:** College of Plant Sciences, Jilin University, Changchun, China

**Keywords:** *T. lanuginosus*, *ANXC7*, mitochondria, conidium development, stress resistance

## Abstract

Annexins (ANXs) are widely expressed and structurally related proteins which play multiple biological roles in animals, plants, and fungi. Although ANXs have been localized to the cytosol and the cell membrane and the molecular basis of the four annexin repeats is well established, the *in vivo* roles of these proteins are still far from clear, particularly with regard to the filamentous fungi. *Thermomyces lanuginosus*, a thermophilic fungus, is widely used in the fermentation industry; however, the role of *ANX* in this organism is unknown. In this study, a single *ANX* homologue (*ANXC7*) was identified and characterized in *T. lanuginosus*. The expression pattern indicated that *ANXC7* is closely associated to conidium development, and it accumulated in the mitochondria of the forming conidia. The deletion of *ANXC7* (*ΔANXC7*) resulted in no obvious phenotype related to colony growth on solid CM medium. However, when *ΔANXC7* was grown in CM liquid culture, the mycelium masses appeared to be larger and looser compared to the wild-type. Additionally, the dry weight of the mutant mycelia was significantly increased. Under conditions that compromise cell-wall integrity, *ΔANXC7* was less vulnerable than the wild-type with regard to such damage. Moreover, based on a surface hydrophobicity test, the *ΔANXC7* strain was clearly less hydrophobic. The growth of *ΔANXC7* was inhibited when grown under selected stress conditions, particularly with regard to salt stress; however, the oxidative resistance to exogenous H_2_O_2_ in *ΔANXC7* was increased, and endogenous H_2_O_2_ levels within the *ΔANXC7* were lower than in the wild-type, thereby suggesting that the *ANXC7* specifically controls oxidative resistance. Based on microscopic observation, 4-day-conidia were more prevalent than 5-day conidia on the conidiophore stalk of *ΔANXC7*, even though the *ΔANXC7* demonstrated an increased production of conidia during these days, indicating precocious conidial maturation and shedding from the conidiophore stalk in this strain. Taken together, our data indicate that *ANXC7* localizes to the mitochondria and is involved in controlling conidium development and oxidative resistance in *T. lanuginosus*.

## Introduction

Annexin is a known calcium (Ca^2+^)-phospholipid binding protein, and the annexin family includes more than 160 unique proteins present in more than 65 different species, ranging from fungi and protists to plants and higher vertebrates (Gerke and Moss, 2002). The principal domains of the annexin protein are the divergent NH_2_-terminal (Hayes et al., 2004) and the conserved COOH-terminal protein core, which harbors a fourfold repeat (I–IV) of 70 amino-acid residues and the Ca^2+^ binding sites. The protein forms a highly α-helical and tightly packed disk with a slight curvature and two distinct sides. In mammals, a number of annexin proteins, including annexin A1, A2, A3, A6, A7, A11, A13, and B7, have been linked to events occurring in the post-trans-Golgi network involved in biosynthetic pathways (Burgoyne, 1988; Creutz, 1992; Raynal and Pollard, 1994; Gerke and Moss, 1997, 2002). In plants, the annexins mediate the homeostatic maintenance of cytosolic free Ca^2+^ and reactive oxygen species within the cellular environment (Gerke and Moss, 2002; Hofmann, 2004; Chasserotgolaz et al., 2005). The majority of annexin proteins in plants are found in the cytosol, though some are found associated with the plasma membrane, endomembranes, or the nuclear envelope (Blackbourn et al., 1992).

Annexin proteins are widely distributed in various fungi, though there is no evidence that this protein exists in *Saccharomyces cerevisiae*, *Candida albicans*, or *Schizosaccharomyces pombe*. The first fungal annexin was identified in the *ascomycete* fungus, *Neurospora crassa*, in 1998 (Braun et al., 1998). At the cellular level, the annexins are highly localized in the cytosol and partly associated with membranes and/or cytoskeleton components (Pratt and Horseman, 1998; Solito et al., 1998). Upon induction of expression in *Aspergillus niger*, *anxc1* accumulates in the cytosol (Khalaj et al., 2004). Co-purification of the annexin homologue in *Saprolegnia* indicates its membrane localization (Moore and Sartorelli, 1992); and *Dictyostelium* expresses two annexin proteins, which localize along the cell membrane and in the cytosol, the nucleus, the endosomal compartment, and the Golgi apparatus (Marko et al., 2006).

Most known functions of the annexin proteins involve their Ca^2+^/phospholipid binding properties and indicate their participation in membrane organization, endocytosis, exocytosis, ion channel modulation, and some extracellular activities (Kourie and Wood, 2000; Gerke and Moss, 2002; Monastyrskaya et al., 2009). The knockout of *anxc1* in *Dictyostelium discoideum* leads to defects in growth, motility, and chemotaxis when the external Ca^2+^ concentration is low, thereby indicating a role for *anxc1* in Ca^2+^ homeostasis. Disruption of the *anxc1* gene in *A. niger* and the *anxc3* gene in *A. fumigatus* reveals a normal growth phenotype and a normal capacity for protein secretion under different conditions. However, a clear role for the annexins, with regard to multiple stress responses in fungi, has been demonstrated in *A. fumigatus*, including the roles in mediating oxidative stress, heavy metal stress, and osmotic stress (Jami et al., 2008; Konopka-Postupolska et al., 2009; Laohavisit et al., 2010; Khalaj et al., 2011).

The thermophilic fungus *T. lanuginosus* can survive at temperatures of 60°C, the highest recorded growth temperature among all eukaryotic species. Based on this special capability, this fungus is widely used in various fields including fermentation, brewing, pharmaceutical production, and waste treatment. As such, research on this fungus has mainly concentrated on a few enzymes, including lipases (Aloulou et al., 2007; Ondul et al., 2012) and xylanases (Singh et al., 2000), as well as physiological and biochemical aspects of its growth. Recently, researchers have advanced the research accessibility of this fungi with the development of *Agrobacterium tumefaciens*-mediated transformation for the generation of insertional mutants in *T. lanuginosus* (Han et al., 2012), leading to the subsequent identification and phenotypic characterization of the *T. lanuginosus MAP1* homolog genes, *PLon* and *MLon* (Cui et al., 2017).

In the current article, a single *ANXC7* protein was confirmed to be mitochondrion-localized in *T. lanuginosus*. The *ANXC7* deletion mutant, *ΔANXC7*, was obtained and biologically analyzed in comparison with the wild-type. *ΔANXC7* increased the production of conidia, but exhibited defects in conidium development and alterations in hydrophobicity. In addition, the enhanced oxidative resistance in *ΔANXC7* was also highlighted in this research.

## Materials and Methods

### Fungal Strains and Plasmids

The *T. lanuginosus* strain 9W, used in this study, was isolated from soil samples in northeast China. All of the wild-type and mutant strains were cultured at 50°C on potato dextrose agar (PDA; 200 g/l peeled potato, 20 g/l glucose, and 16 g/l agar) or complete minimal media [CM; 1 g/l yeast extract, 0.5 g/l casein enzymatic hydrolysate, 0.5 g/l casamino acids hydrolysate, 10 g/l glucose, 1 g/l Ca(NO_3_)_2_⋅4H_2_O, 0.2 g/l KH_2_PO_4_, 0.25 g/l MgSO4⋅7H_2_O, 0.15 g/l NaCl, and 16 g/l agar].

The *A. tumefaciens* strain AGL-1 was used for the transformation of the *T. lanuginosus* strain. Two plasmids were employed in this study. One of them was the PXEH vector containing the upstream and downstream flanking regions of the target gene, allowing for gene-specific deletions. This vector also conferred kanamycin resistance in *A. tumefaciens* and *E. coli* and harbored the hygromycin B phosphotransferase (hyg) gene as a selection marker for the *T. lanuginosus* deletion mutants. The other vector used was the pKD7-RED vector (donated by Wang Hongkai and Jianping Lu, Zhejiang University), which contained the *DsRED* gene as a subcellular localization tag and harbored a G418-resistance gene as a selection marker.

To construct the *ANXC7* deletion mutant, the left-border (LB) and right-border (RB) flanking sequences of the *ANXC7* gene were PCR-amplified using both primer pairs of ANXC7-LB-F/-LB-R and ANXC7-RB-F/-RB-R (**Supplementary Table S1**) and the wild-type genomic DNA template previously extracted by CTAB method. The two flanking regions were ligated to both sides of the hygromycin B phosphotransferase resistance gene in the PXEH vector (**Supplementary Figure S1A**). The constructed vector was then used to generate the *ΔANXC7* mutant strain (**Supplementary Figure S2**).

To ensure that the obtained mutant phenotype could be attributed to the desired deletion and to investigate the sub-localization of the protein, *ΔANXC7* was complemented by integration of the wild-type *ANXC7* gene to generate the complementation strain *Canxc7* (**Supplementary Figure S2**). The PKD7-RED-ANX vector for complementation was constructed by extracting RNA from *T. lanuginosus* 9W and cloning the coding sequence of *ANXC7* into the upstream of *DSRED2* in the PKD7-RED vector (**Supplementary Figure S1B**). This complementation vector containing the fusion gene of *DsRED2-ANXC7* for localization analysis was then transferred into *ΔANXC7*.

All primers were designed using DNAMAN 8 (Lynnon Biosoft, United States). High fidelity fusion polymerase (Fermentas, United States), SYBR Premix Ex Taq, restriction enzymes, T4 DNA ligase, and other DNA-modifying enzymes were used as recommended by the supplier (TaKaRa, Dalian, China).

### *Agrobacterium tumefaciens*-Mediated Transformation (ATMT)

The transformation process was modified based on a prior study (Khan et al., 2014). *A. tumefaciens* AGL-1, carrying a deletion or expression vector, was cultured at 28°C in 10 ml Luria-Bertani medium (LB), with 50 μg/ml rifampicin and 50 μg/ml kanamycin, overnight in a rotatory shaker (180 rpm). Subsequently, ∼2.0 ml of the culture was centrifuged at 2,400 *g* for 10 min, and the precipitate was resuspended in inducible medium supplemented with 200 μM acetosyringone (AS) to achieve an optical density (OD) between 0.2 and 0.4 at 600 nm, as assessed using a microplate reader (Molecular Devices, Sunnyvale, CA, United States). The medium was cultured with agitation at 180 rpm and 28°C for 8–10 h until an OD600 nm value of 0.8 was reached. *T. lanuginosus* 9W was cultured on PDA at 50°C for 4–5 days. The conidia were then washed from the clones using induction medium (IM, LB containing 100 μM acetosyringone) and adjusted to a final concentration of 1 × 10^5^ conidia/ml.

Sterile Hybond N membranes (Amersham Biosciences, Piscataway, NJ, United States) were placed on solid IM + AS plates. An equal volume of the corresponding concentration of the *T. lanuginosus* conidia was mixed with AGL-1 culture and 100 μl was pipetted onto the filters. The plates were co-cultured at 28°C for 2 days. The filters were then transferred onto selective PDA medium containing 80 μg/ml hygromycin B at 50°C in the dark until colonies appeared. Expression transformants were selected with 50 μg/ml G418 instead of hygromycin B.

### Bioinformatic Analysis

Nucleotide and protein sequences were searched from the Fungal Genomes Database^1^ and analyzed using PubMed online tools and DOG 2.0 – Protein Domain Structure Visualization^2^; and the schematic diagram of the protein was constructed using IBS 1.0.1 software. Multiple sequence alignment was performed with the selected annexin protein sequences using DNAMAN 8 (Lynnon Biosoft, United States). The spatial structure of *ANXC7* was predicted with the SWISS-MODEL server^3^ and the structure diagram was generated with Swiss-PdbViewer 4.1.0. A phylogenetic tree was established via the neighbor-joining tree available in MEGA7.0.9. To analyze the sublocalization of *ANXC7*, the online analysis platform^4^ was used; and the Target Signal Predictor analysis of the sequence of *ANXC7* was performed at the website of http://www.cbs.dtu.dk/services/TargetP/ to predict the mitochondrial transit peptide (mTP).

### Fluorescence Microscopy

In order to observe the subcellular localization of the *ANXC7* protein, C*anxc7* was cultured in a PDA plate in which the coverslips were obliquely inserted into the culture medium for 5 days in the dark and observed using fluorescence microscopy until the mycelium extended to the coverslips. Mycelia were harvested from the PDA plate, washed twice with dd water, and incubated with 100 nM Mito-green for 30 min. Mito-Green is a carbocyanine-based and mitochondrion-selective green fluorescence regent (Invitrogen, Ltd., Paisley, United Kingdom), which can be used to dye and then detect or track the presence of the mitochondrion location when it is excited by the 488 nm laser wavelength. Mito-green and DsRED2 fluorescence were observed under an Olympus Xa21 microscope (Olympus, Tokyo, Japan).

### Quantitative Real-Time PCR (qRT-PCR)

The conidia suspension was incubated in CM at 50°C for 4 days in a rotatory shaker at 180 rpm. Mycelia were then leached, and TRIzol was used to extract the total RNA (TaKaRa, Dalian, China). First strand cDNA was synthesized using an oligo (dT) primer from total RNA, which was treated with DNase I. Subsequently, qRT-PCR was performed using an ABI7500 System and SYBR Premix Ex Taq (TaKaRa, Dalian, China). The relative mRNA levels were calculated using the 2^-ΔΔC_t_^ method. The actin gene was used as an internal standard. The primer sequences used for qRT-PCR are listed in supplementary information (**Supplementary Table S1**).

### Mycelial Form and Mycelial Dry Weight Determinations

To observe the morphological changes of the mycelium, a conidia suspension was added to the CM liquid medium and incubated at 50°C for 7 days in a rotatory shaker at 180 rpm. To determine the biomass of wild-type and *ΔANXA7*, 1 ml of spore suspension containing 1 × 10^5^ conidia was incubated with 100 ml of CM liquid media at 50°C for 7 days. The collected mycelium was then washed twice using ddH_2_O and desiccated in an oven. Each assay was independently repeated three times.

### Cell-Wall Integrity Test

The cell-wall integrity test was conducted by growing a 5-mm mycelial plug of wild-type 9W or mutant strain on CM plates containing Congo red (0.2 or 0.3%; w/v) or SDS (0.005 or 0.01%; w/v). Both of these compounds interfere with the fungal cell-wall assembly (Woldringh and Van Iterson, 1972). The plates were incubated for 7 days in the dark at 50°C. The diameters of the fungal colonies were photographed and measured 7 days after inoculation. This experiment was performed in triplicate and repeated three times for each strain.

To assess the influence between the stress responses to simulated stress conditions caused by the gene deletion, different concentrations of NaCl (2.5 and 5%), CaCl_2_ (2.5 and 5%), or sorbitol (2.5 and 5%) were added to the CM solid media, respectively. The mycelial plug of wild-type and mutant strains was then inoculated on the corresponding stress medium at 50°C. The diameters of the fungal colonies were photographed and measured 7 days after inoculation. This experiment was performed in triplicate and repeated three times for each strain.

### Hydrophobic Experiments

In order to observe the change in hydrophobicity of the mycelium, we prepared wild-type and *ΔANXA7* colonies that had been cultured at 50°C for 5 days. A total of 10 μl of different solutions were gently placed in a consistent position in the middle stage of the colony growth. These solutions included distilled water, SDS, ethylene diamine tetraacetic acid (EDTA), Tween 20, and 0.2% gelatin. The experimental plates were placed at room temperature for 4 h and then observed and photographed. This experiment was performed in triplicate and repeated three times for each strain.

### H_2_O_2_ Treatment and Other Stressors

To investigate the effects of exogenous oxidative stress on the wild-type and Δ*ANXC7* strains, each strain was cultured on CM agar containing 2.5 or 5 mM H_2_O_2_ for 7 days at 50°C. Osmotic stress conditions were induced by supplementing the CM agar with NaCl and sorbitol at final concentrations of 2.5% (w/v) and 5% (w/v), respectively. The diameters of the fungal colonies were photographed with a Canon DS126231 digital camera and measured 7 days after inoculation. This experiment was performed in triplicate and repeated three times for each strain.

### Endogenous H_2_O_2_ Measurements

The H_2_O_2_ content was determined as previously described for plants (Brennan and Frenkel, 1977). Hydrogen peroxide (H_2_O_2_) was extracted by homogenizing 3 g of mycelia from the wild-type or *ΔANXC7* strains in 6 ml of cold acetone. The homogenate was then centrifuged at 3500 *g* for 5 min at room temperature, and the resulting supernatant was designated as the sample extract.

Thereafter, 0.1 ml of titanium reagent [5% (w/v) titanic sulfate in concentrated H_2_SO_4_] was added to 1 ml of the sample extract, followed by the addition of 0.2 ml of strong aqueous ammonia to precipitate the peroxide-titanium complex. The precipitated sample was centrifuged at 3000 *g* for 10 min at room temperature, the supernatant was discarded, and the precipitate was then solubilized in 5 ml of 2 M H_2_SO_4_. The absorbance of the samples was determined at 415 nm against a 2 M H_2_SO_4_ blank. The H_2_O_2_ concentration in the samples was determined by comparing the absorbance against a standard curve of 0–5 μM titanium-H_2_O_2_ complex. This experiment was performed in triplicate and repeated three times for each strain.

The measurement process was modified based on a prior study (Brennan and Frenkel, 1977). First, a standard curve was prepared according to Cui et al. (2017) by adding 0–5 μM titanium–H_2_O_2_ complex to seven 15 ml centrifuge tubes. After a 300 rpm centrifugation for 10 min, the supernatant was discarded. A total of 6 ml of 2 M sulfuric acid was then added to each tube to dissolve the precipitate, and the absorbance of each tube was detected at 415 nm (**Supplementary Figure S3**). Subsequent experiments were performed in triplicate and repeated three times for each strain.

### Premature Conidial Growth and Growth Rate Assays

We determined whether the conidial grown was premature based on the results of conidial production and conidial germination rate assessments. First, the conidial production was assessed by growing a 5-mm mycelial plug of the wild-type and mutant strains on PDA plates at 50°C. The conidia of the strains were counted and washed with distilled water every 24 h. New PDA plates, in which a sterilized cover slip had been inserted in advance, were then prepared with wild-type and mutant mycelial plugs. The plates were then placed in a 50°C incubator and observed every 24 h. For observation, the cover glass was gently removed with forceps and placed on an optical microscope. This experiment was used to observe the morphology of the conidia and was performed in triplicate and repeated three times for each strain.

To measure the differences in conidial germination between the wild-type and mutant strains, 100 μl of conidial suspension (10^4^ conidial/ml) was mixed with 100 μl PDB (PDA medium without agar), added to each well of a 96-well plate, and then incubated at 50°C. After 2, 4, 6, 8, and 12 h, the corresponding experimental group was placed on a microscope slide and observed using a Nikon YS100 microscope (Nikon, Japan). Each assay was independently repeated three times for each strain.

### Statistical Analysis

All experiments were repeated at least three times. The means ± SD of the colony diameter, germination rate, and relative expression were determined using GraphPad Prism 7.00 software. Error bars represent standard deviation. Data were analyzed using InStat3. *P* < 0.05 was considered statistically significant.

## Results

### The ANX Homologue in *T. lanuginosus*

**FIGURE 1:**
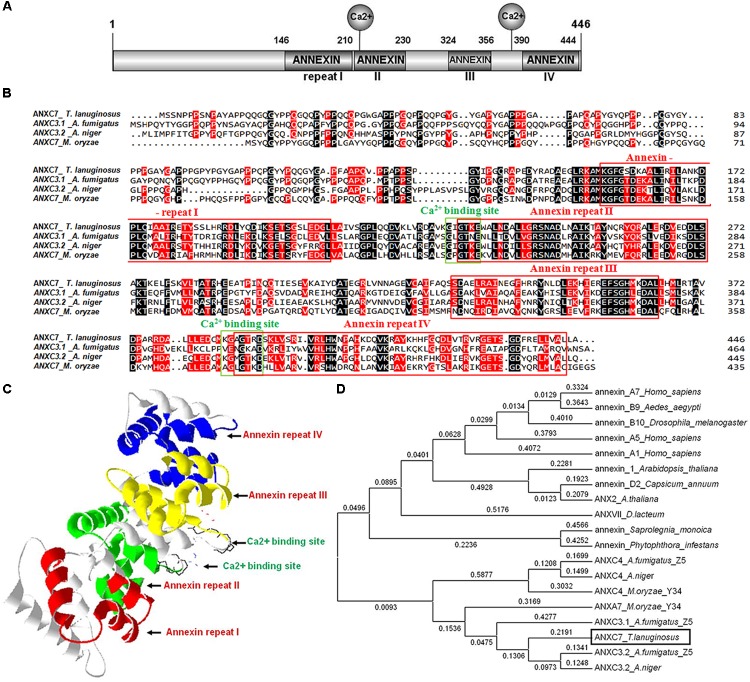
The *ANXC7* gene (*ANXC7*) of *T. lanuginosus*. **(A)** A schematic representation of the domain architecture of the *T. lanuginosus* annexin gene is shown indicating the main specificity domains, four annexin repeats, and two Ca^2+^ binding sites. **(B)** Protein sequence alignment. The four red boxes are four annexin repeats; the two green boxes are two Ca^2+^ binding sites; besides *ANXC7*, the other three ANXs are *Aspergillus fumigatus* *ANXC3.1*, *Aspergillus niger ANXC3.2*, *Magnaporthe oryzae ANXC7* (XP_003709567), respectively. **(C)** The spatial structure prediction of 27 AgGlpF protein using SWISS-MODEL server, illustrating the highly helical folding of the protein core that forms a slightly curved disk; different colors were chosen to highlight the four annexin repeats: red (repeat I), green (repeat II), yellow (repeat III), and blue (repeat IV). **(D)** Phylogenetic tree based on the annexin C7 protein domain from different eukaryotic organisms indicating that *ANXC7* has a relatively close relationship with the fungi group.

The annexin protein involves two principal domains: the divergent NH_2_-terminal and the conserved COOH-terminal. The latter domain contains the major conserved structural region, the so called annexin repeat, which consists of approximately 70 amino acid residues and Ca^2+^ binding sites (Khalaj et al., 2015). Based on the conserved amino acid sequence of the *A. fumigatus, A. niger*, and *Magnaporthe oryzae* annexin proteins, a unique annexin homolog Thela2p4_001773 (Accession Number: MH553929) was identified in the *T. lanuginosus* genome^1^. The protein encoded by the Thela2p4_001773 gene contained the two principal domains described above. The total length of the Thela2p4_001773 gene was 1401 bp, including one intron, two exons, and a 1341 bp coding region, which encoded a putative 466 amino acid protein. For the Thela2p4_001773 protein, four annexin repeats were identified at amino acid positions 157–210, 230–279, 329–365, and 390–444 (**Figures 1A,B**). In addition, the Ca^2+^ binding sequence GIGTKE, which normally participates in the formation of type II Ca^2+^ binding sites (Geisow et al., 1986), was located at the amino acid position 228. Additionally, a GAGTR Ca^2+^ binding sequence was identified at the amino acid position 388 (**Figure 1B**). The deduced 3D structure of the Thela2p4_001773 protein indicated that the four annexin repeats were packed into one α-helical disk with the Ca^2+^ binding sites present on the convex surface of this structure (**Figure 1C**).

The amino acid sequence of Thela2p4_001773 was aligned and compared with several reported sequences of annexin proteins. Thela2p4_001773 showed 64% similarity with ANXC3.2 from *A. niger* and 44% similarity with ANXC3.1 from *A. fumigatus* (Khalaj et al., 2004). Phylogenetic analyses of Thela2p4_001773 within the eukaryotic tree indicated that this protein was closely related to the other fungal annexin proteins classified as group C (**Figure 1D**). As such, the Thela2p4_001773 protein was termed as *ANXC7*.

### ANXC7 Is Localized in the Mitochondria of Developing Conidia

**FIGURE 2:**
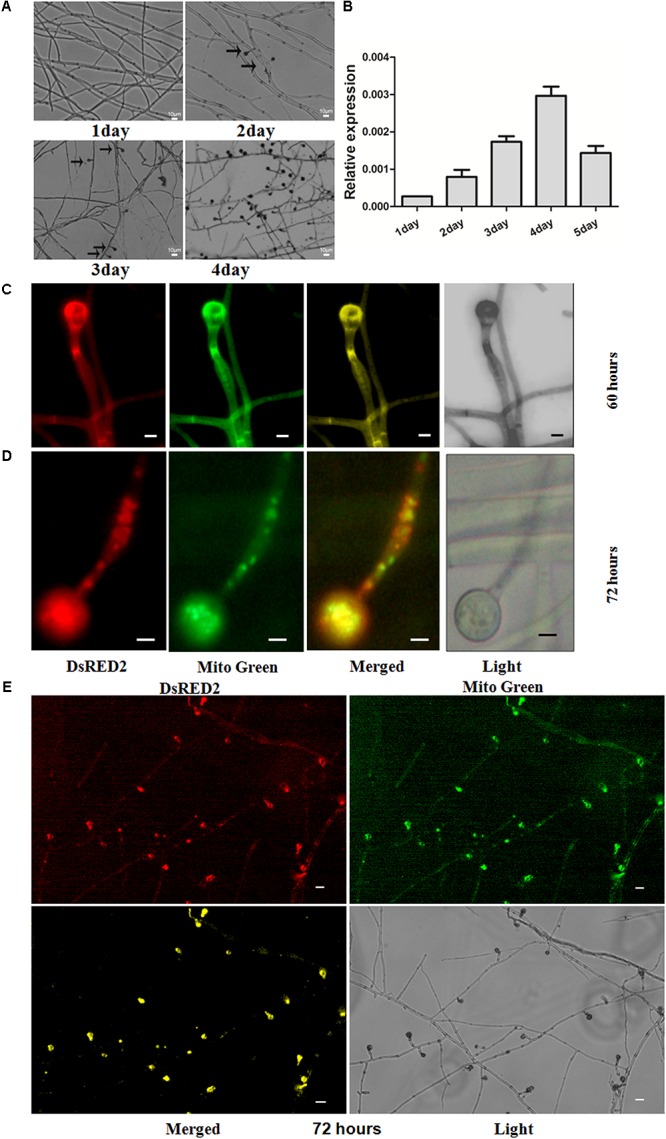
Expression patterns of *ANXC7* gene and intracellular localization of ANXC7 protein. **(A)** Development of *T. lanuginosus*, from growth to reproduction. The arrow indicates to the conidium. Bar = 10 μm. **(B)** The relative expression of *ANXC7* gene during different time points. The data represent means ± standard deviations (SD) of three experiments. **(C–E)** Confocal laser scanning microscopy analysis of the subcellular localization: in the merged image, the original red fluorescence representing the *ANXC7-DsRed* protein was coincident with the green fluorescence of Mito-green (green fluorescent dye as a membrane marker), appearing as yellow areas; Images were acquired after 60 h **(C)** and 72 h **(D,E)** of growth in PDA medium. Bar = 2 μm.

The life cycle of the thermophilic fungus *T. lanuginosus* is comprised of a vegetative mycelium growth stage and a conidial reproductive stage (Cui et al., 2017). In the CM liquid medium (50°C), the mature conidia immediately began to germinate into mycelium, and the next generation conidia sporadically appeared on the second day. On the fourth day, a large number of mature conidia, which were investigated with the microscope observations, were able to germinate and enter the next stage in the lifecycle (**Figure 2A**).

The expression profile of *ANXC7* was analyzed at different culture times using the qRT-PCR technique. The thermophilic fungus was inoculated in parallel on identical CM plates. The total mRNA was then extracted at five time points (i.e., 1, 2, 3, 4, and 5 days). The result showed that the *ANXC7* gene was expressed at a low level in the early stage of mycelium growth (**Figure 2B**), which is similar to the members of the *ANXC* family in *Dictyostelium annexins* (Marko et al., 2006). With the appearance of conidia on the second day, the *ANXC7* gene expression accordingly increased and reached a maximum on the fourth day (**Figure 2B**), which rightly coincided with the time dynamics of conidia from growth to maturation (**Figure 2A**), suggesting its association with conidia development.

The *ANXC7* deletion mutant, *ΔANXC7*, was generated with targeted gene replacement. Then, based on the *ΔANXC7* strain, we created the complementation strain (*Canxc7*) (**Supplementary Figure S2**). Under normal growth conditions, the *ΔANXC7* was almost consistent with the *Canxc7* and wild-type strains (**Supplementary Figure S2A**), in spite of the high levels of expression of *ANXC7* in *Canxc7* and no expression in the wild-type (**Supplementary Figures S2B,C**).

Based on the online target signal predictor (Query Protein: nucl: 10.5, cyto: 7.5, mito: 4; and mTP score is 0.109), the *ANXC7* protein probably localizes onto the mitochondrion. We then applied the complementation strain of *Canxc7* for *in vivo* investigation. In the *Canxc7* strain, the *ANXC7* and *DsRED* genes were fused, which was mainly used to determine the subcellular location of the *ANXC7* protein (**Figures 2C–E**). After 60 h of cultivation, we observed that DsRED fluorescence co-localized with Mito-Green fluorescence at the cell membrane, and this was most apparent during the conidia formation, which presented with clear apex enrichment of these fluorescence signals (**Figure 2C**). As the incubation time was extended to 72 h, the *ANXC7* protein appeared to be localized to the mitochondria of conidia and the top of conidiophores (**Figure 2D**), thereby suggesting that the *ANXC7* protein primarily accumulated in the mitochondria of forming conidia (**Figures 2D,E**).

### *ANXC7* Loss Enhances the Growth of Mycelia in Liquid Culture

**FIGURE 3:**
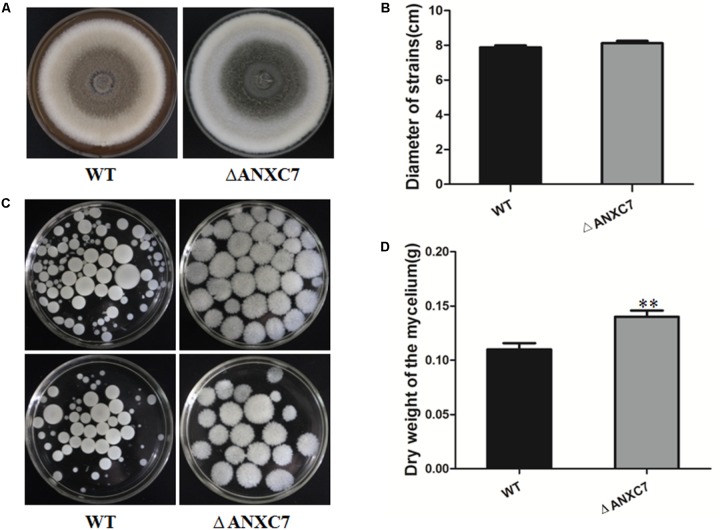
Colony morphology and dry cell weight of the wild-type and *ΔANXC7* strains. **(A)** The growth of the wild-type and *ΔANXC7* mutant strains on PDA medium at 50°C for 7 days. **(B)** The colony diameter data from the wild-type and *ΔANXC7* mutant strains grown on PDA medium at 50°C for 7 days. **(C)** The conidia suspension was added to CM liquid medium and incubated at 50°C for 7 days in a rotatory shaker at 180 rpm. **(D)** Mycelial dry weight of *ΔANXC7* mutants in CM media when grown at 50°C with shaking (180 rpm) for 7 days. *WT*: wild-type, Δ*ANXC7*: *ANXC7* mutant. The data represent means ± SD of three experiments. Error bars represent standard deviation. Significant differences between the wild-type strains and the mutant strains are indicated as ^∗∗^*p* < 0.01.

The wild-type and *ΔANXC7* strains were, respectively, inoculated on the CM solid medium. As described above, the *ANXC7* deletion mutant grew at a slightly increased rate compared with the wild-type after culturing at 50°C for 7 days (**Figures 3A,B**). Strangely but interestingly, when both the wild-type and the *ΔANXC7* strains were inoculated in CM liquid medium at 50°C for 7 days, most of the mycelium masses of the *ΔANXC7* strain appeared to be looser and larger in comparison with the wild-type strain (**Figure 3C**); in addition, the dry weight of the *ΔANXC7* strain was significantly increased, reflecting an enhanced growth of *ΔANXC7* mycelia in liquid CM culture (**Figure 3D**).

### *ANXC7* Is Important for the Hydrophobicity of Colony Surface

**FIGURE 4:**
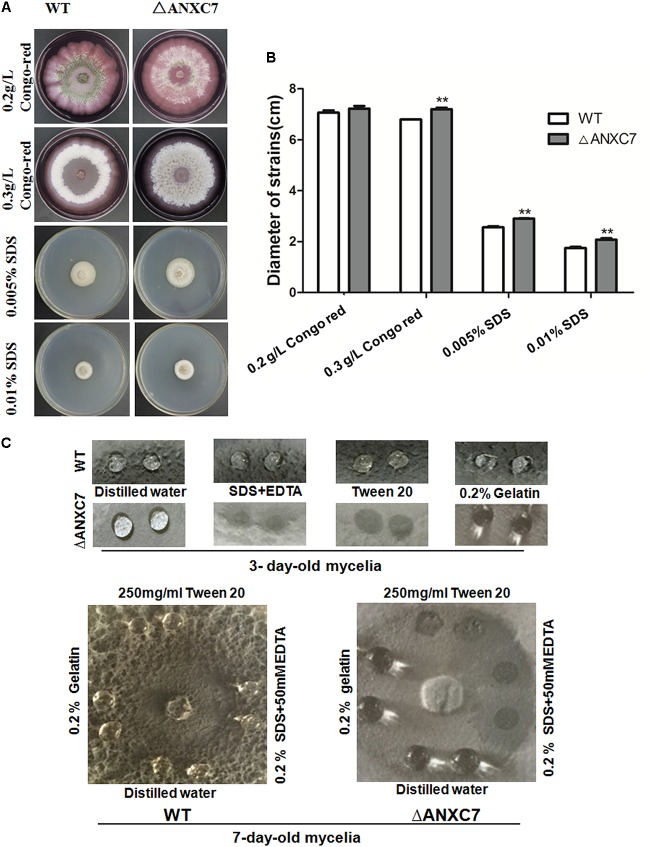
Wild-type and *ΔANXC7* cell-wall integrity and surface hydrophobicity tests. **(A)** The strains were cultured in different concentrations of Congo red or SDS medium for 7 days at 50°C. **(B)** Under the conditions of compromised cell-wall integrity, there were no significant differences at *P* < 0.01, though the colony of the mutant (the asterisk ^∗^ indicated) appeared to be larger than that of the wild type. **(C)** Surface hydrophobicity assays. A 10 μl drop of H_2_O, 250 mg/ml Tween 20, 0.2% SDS+50 mM EDTA, or 0.2% gelatin was placed on 3-day-old (above) and 7-day-old (below) cultures. Drops of Tween 20, 0.2% and SDS+50 mM EDTA remained on the cultures of wild-type, while they were soaked into the *ΔANXC7* colonies. The photographs were taken after a 4 h incubation.

Biochemically, the annexins are known to bind to negatively charged phospholipids in a Ca^2+^-dependent manner and to participate in various physiological activities related to cellular membranes. To assess the effects of Δ*ANXC7* on cell-wall integrity, we cultured the wild-type and mutant strains on CM agar supplemented with 0.2 g/l or 0.3 g/l Congo red. After 7 days, the average diameter of the tested colonies was 7.14 cm and 7.43 cm, respectively, for the wild-type and mutant strains grown on the 2 g/l Congo red medium, and was 6.85 and 7.34 cm for the two strains grown on CM supplemented with 3 g/l Congo red. Therefore, there is no significant difference in the growth between the wild-type and mutant strains (**Figures 4A,B**). When the wild-type and mutant strains were cultured on a CM agar plate containing SDS (0.01%, 0.05%), a similar result was obtained (**Figures 4A,B**), suggesting that *ANXC7* loss is incapable of affecting the cell-wall integrity under corresponding treatments.

Prior reports have suggested that the annexin interacts with membrane phospholipids to influence the hydrophobicity of the plant surface of the bell peppers (*Capsicum annuum*) (Dabitz et al., 2005; Talukdar et al., 2009). Fungal surface hydrophobicity analysis between the wild-type and mutant strains was performed. We dripped drops of sterile distilled water, SDS + EDTA, Tween 20, or 0.2% gelatin on the colony surface of these strains after culturing them at 50°C for 3 or 7 days. At 4 h we observed no obvious change in the wild-type strain. Conversely, the SDS + EDTA and Tween 20 droplets on the *ΔANXC7* strain were soaked (**Figure 4C**), indicating that the lack of the *ANXC7* gene decreased the surface hydrophobicity of the fungi, allowing the droplets to be absorbed.

### Resistance to H_2_O_2_ Increased but Resistance to Osmotic and Salt Stress Decreased, in *ΔANXC7*

**FIGURE 5:**
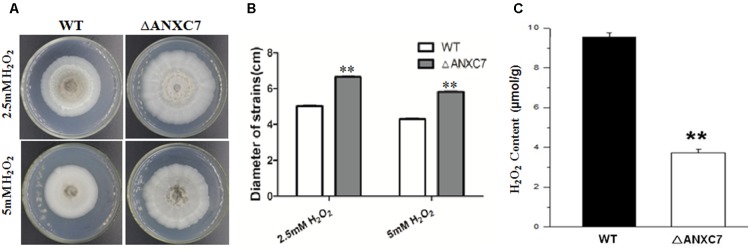
*ΔANXC7* strains are more sensitive to oxidative stress, and accumulate less H_2_O_2_ than the wild-type. **(A)** The *ΔANXC7* strain was less affected than the wild-type when both were inoculated on CM containing 2.5 or 5 mM H_2_O_2_. **(B)** Under the conditions of 2.5 or 5 mM H_2_O_2_, the measured colony diameters of the *ΔANXC7* mutant and the wild-type. **(C)** H_2_O_2_ concentration of the mutant and wild-type strains. The data represent means ± SD of three experiments. Error bars represent standard deviation. Significant differences between the wild-type strains and the mutant strains are indicated as ^∗∗^*p* < 0.01.

Some members of the annexin family have stress-related functions (Talukdar et al., 2009). To address the relationship between the *ANXC7* gene and oxidative stress in *T. lanuginosus*, the wild-type and mutant strains were cultured on CM agar containing 2.5 mM or 5.0 mM H_2_O_2_ at 50°C for 7 days. The result indicated that the wild-type strain was more greatly affected, but the *ΔANXC7* strain was less affected when H_2_O_2_ medium was added into CM agar (**Figures 5A,B**), suggesting the growth-inhibitory effect of *ANXC7* under oxidative stress.

The endogenous H_2_O_2_ in each strain was measured using the determination of absorbance at 415 nm, as based on previously published methods (Brennan and Frenkel, 1977). The result indicated that less H_2_O_2_ accumulated in the *ΔANXC7* strain: the H_2_O_2_ accumulated in the *ΔANXC7* mycelia was approximately two-fifths of that in the wild-type (**Figure 5C**). Linking both the above results, and considering the pleiotropic effects of H_2_O_2_ on cell growth, the enhancement of oxidative resistance in *ΔANXC7* should be the direct or indirect result of reduction of the endogenous H_2_O_2_.

**FIGURE 6:**
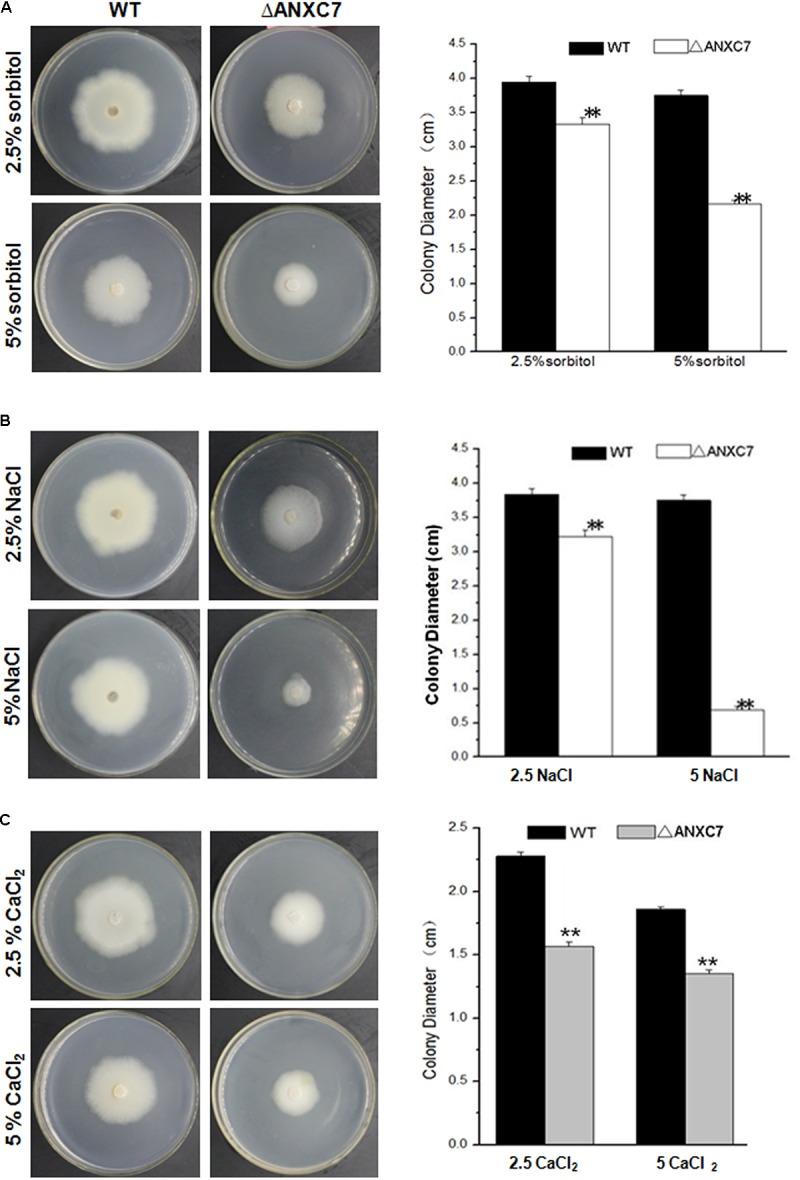
*ΔANXC7* strains are more sensitive to osmotic and salt stressors than the wild-type. Colony growth comparisons of the *ΔANXC7* and wild-type strains on CM media with **(A)** sorbitol (2.5 and 5%), **(B)** NaCl (2.5 and 5%), and **(C)** CaCl_2_ (2.5 and 5%) at 50°C for 4 days, respectively. The data represent means ± SD of three experiments. Error bars represent standard deviation. Significant differences between the wild-type strains and the mutant strains are indicated as ^∗∗^*p* < 0.01.

To check if similar phenomena occur under conditions of osmotic and salt stress, the wild-type and *ΔANXC7* were, respectively, cultured on CM agar supplemented with sorbitol, NaCl, and CaCl_2_ at 50°C. Four days later, the colony growth levels of the wild-type and deletion mutant strains were measured. In comparison with the wild-type, however, the *ΔANXC7* strain appeared to be much more sensitive under stress environment. When 5% of sorbitol or NaCl was added into complete agar medium, the growth of hyphae in the *ΔANXC7* strains was seriously inhibited, in spite of the observation that the wild-type strain was also slightly affected (**Figures 6A,B**). Similarly, when NaCl (2.5%) was replaced with CaCl_2_ (2.5%), the *ΔANXC7* strain was also inhibited; however, with the increase of concentrations of CaCl_2_ to 5%, the *ΔANXC7* strain was almost no longer inhibited compared with itself in 2.5% concentrations (**Figure 6C**). This difference between the sensitivity to the oxidative stress and to the osmotic and salt stress suggests that the *ANXC7* gene participates in different regulation mechanisms under different stresses, and even the resistance mechanisms to Ca^2+^ and Na^+^ mediated by *ANXC7* are different.

### *ANXC7* Is Involved in Controlling Conidium Development

**FIGURE 7:**
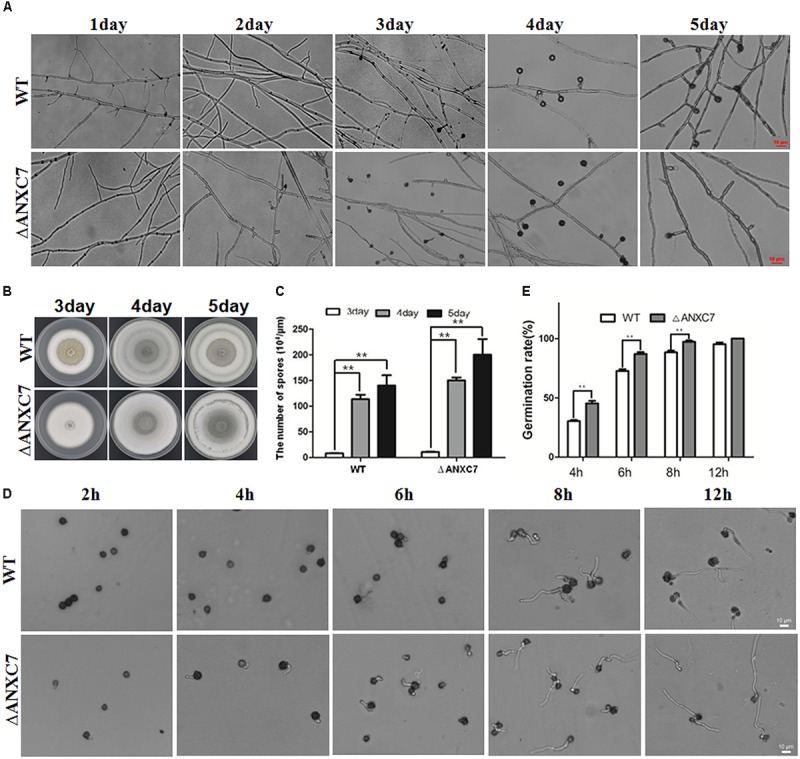
Premature and rapid growth of *ΔANXC7*. **(A)** Under a microscope, mycelium growth and spore formation were observed. The *ΔANXC7* spores appeared on the second day; and a large number of emptier conidiophore stalks (without conidia) in *ΔANXC7* gradually formed from the fourth to fifth day. **(B)** The growth of the wild-type and *ΔANXC7* strains on PDA at 50°C for 5 days; pictures were taken every 1 h. **(C)** The amount of conidia of the wild-type and *ΔANXC7* mutant increased from the third to fifth day. **(D)** Spore germination was observed and photographed every 2 h. The spore suspension cultured in PDA liquid medium at 50°C. **(E)** Spore germination rate of the wild-type and *ΔANXC7* strains. The data represent means ± SD of three experiments. ^∗∗^indicates a very significant difference at *P* < 0.01. Error bars represent standard deviations.

Based on the above investigations, the absence of the *ANXC7* gene did not cause any obvious phenotypic changes associated with hyphae growth under normal mediation conditions. We investigated sporogenesis and conidia germination of the tested strains. The wild-type strain and *ΔANXC7* were, respectively, inoculated on a block of PDA agar. The block of agar was then placed on a glass slide and cultured at 50°C. With the growth of colonies, the extended mycelia climb onto the cover glass. Based on microscopic observation, these two strains began to produce conidia from the second day, and a large number of mature conidia were observed on the fourth day (**Figures 2A**, **7A**); however, many emptier conidiophore stalks (with less or no conidia) gradually appeared in *ΔANXC7* in the fourth and fifth days (**Figure 7A**); however, many emptier conidiophore stalks (with few or no conidia) in *ΔANXC7* gradually appeared from the fourth to fifth day (**Figure 7A**) despite the fact that the amount of conidia increased day by day, whether they were the wild type or the mutant strains, while all conidia on the plate were collected and counted (**Figures 7B,C**), implying that a correlation between the abscission of conidia and the deletion of *ANXC7* gene.

Conidial germination was also investigated and analyzed between the wild-type and mutant strains. The tested conidia were collected from the fourth day solid media, then statically inoculated in the PDA liquid medium at 50°C, and finally observed and counted every 2 h. The results indicated that the *ΔANXC7* strain started germinating from the fourth hour, but the wild-type started from the sixth hour (**Figure 7D**). The conidial germination rate of the mutant was observed to be significantly higher than that of the wild-type (**Figures 7D,E**). On the whole, the *ANXC7* gene played an important role in the germination, growth, maturation, and abscission of the conidium in *T. lanuginosus*.

## Discussion

A large number of ANXs have been characterized in the plant and animal cells. Moreover, many studies have confirmed that the ANXs can act as cytosolic, peripheral, and even integral membrane proteins depending on the cellular pH, cytoplasmic Ca^2+^ concentrations, membrane oxidation levels, lipid composition, and voltage. By comparison, little is known about the ANXs of filamentous fungi, though a number of *ANXs* have been described in *Ascomycetes*, *Basidiomycetes*, and *Oomycetes* (Khalaj et al., 2004; Moss and Morgan, 2004). In this report, we genetically and biologically analyzed the *ANXC7* gene in the thermophilic fungus *T. lanuginosus*.

The genes of *ANXs* express in either a constitutive or a highly inducible pattern, and generally play important roles in the cytosol, cytomembranes, or cytoskeleton components (Pratt and Horseman, 1998; Solito et al., 1998; Brachvogel et al., 2003; Herr et al., 2003; Khalaj et al., 2015). The expression pattern of the *ANXC7* gene was closely associated to the conidial development (**Figure 2A**), suggesting its inducible characteristics.

We have tried several methods to predict certain destination signals in *ANXC7*. By analyzing online on the website of https://wolfpsort.hgc.jp/, the protein was predicted to be localized onto three organelles including the mitochondrion (Query Protein: nucl: 10.5, cyto: 7.5, mito: 4), thereby suggesting the existence of a certain relatively conserved peptide signal. In addition, when we predicted the signal in the website http://www.cbs.dtu.dk/services/TargetP/, a putative mitochondrial transit peptide (mTP) was predicted to be 0.109. According to the notes about scores in the website, the location with the highest score is the most likely according to TargetP, and the relationship between the scores may be an indication of how certain the prediction is. Indeed, the subcellular location of the *ANXC7* protein was confirmed using a confocal laser scanning microscope. It was localized onto the mitochondria of forming conidia (**Figures 2C–E**), indicating the unique subcellular location of the ANXC7 protein, and which is obviously different from the most characterized ANXs such as ANXC1A and ANXC1B (Morgan et al., 2004).

Annexins can reversibly bind to negatively charged membrane phospholipids in the presence of micromolar cytoplasmic Ca^2+^ concentrations; and Ca^2+^-dependent binding to negatively charged acidic membrane phospholipids is a landmark characteristic of the annexin protein family due to its evolutionary-conserved structural motifs (Geisow et al., 1986). The presence of a hydrophilic pore at the center of the molecule is proposed to be the structural basis for the annexin Ca^2+^ channel activity (Gerke and Moss, 2002). In this research, the *ANXC7* gene encodes a protein sequence consistent with the particular structure of the annexin family, including the two principal domains and Ca^2+^ binding sites (**Figure 1**). Above all, the *ANXC7* fusion protein accumulated at the tip of the forming conidium (**Figure 2C**), and the *ΔANXC7* strain was no longer further inhibited with the increase of Ca^2+^ concentrations (**Figure 6C**). Thus, we deduced that the ANXC7 protein belongs to one of the Ca^2+^-binding proteins given the fact that the developing fungal mycelia or plant tissues have a high concentration of Ca^2+^ at their distal or tip ends (Konopka-Postupolska et al., 2011; Kim et al., 2012; Khalaj et al., 2015).

In plants, the surface hydrophobicity is altered in the annexin mutants due to hydrophobic interactions between the annexin proteins and membrane phospholipids, as demonstrated in bell pepper (*Capsicum annuum*) plants, in which AnnCa32 interacts with membrane phospholipids via hydrogen bonding of several amino-acid residues to the phospholipid head group and glycerol backbone (Talukdar et al., 2009). Similar to the case in plants, the *ANXC7* gene deletion led to less hydrophobicity according to the surface hydrophobicity test in this research, indicating that the *ANXC7* gene plays an important role in maintaining the surface hydrophobicity in *T. lanuginosus*.

*Aspergillus fumigatus Rod* gene (U06121) that encodes the conidial hydrophobin is associated with the surface hydrophobicity (Paris et al., 2003). Based on the Rod protein sequence, we searched two *Rod*-similar genes (*Thela2p4_006088* and *Thela2p4_006090*) in *T. lanuginosus* genome. Using the qRT-PCR technology, we found that the expression levels of the *Thela2p4_006088* (or *Thela2p4_006090*) between the mutant and wild-type strains were not different, therefore we preliminarily thought that the surface hydrophobicity alteration in our deletion mutant is not associated with the Rod. However, as noted above, the *ANXC7* gene loss is incapable of affecting the cell-wall integrity under SDS and Congo red conditions (**Figure 4A**). Interestingly, the growth of *ΔANXC7* was inhibited when the mutant strain grew under selected stressors, particularly with regard to salt stress (**Figure 6**). This result suggested that the *ANXC7* protein provides a direct or indirect benefit to the membrane by protecting the surface characteristics against hydrophobicity alterations or by controlling the permeability of membrane barrier under salt conditions.

The annexins were proposed to function as candidate Ca^2+^ channels involved in responding to ROS, as the annexins were able to promote ROS-stimulated passive Ca^2+^ transport (Laohavisit et al., 2010). Elevation of cytoplasmic free Ca^2+^ is a regulatory step involved in immunity resistance, stress adaptation, and development process (McAinsh and Pittman, 2009). On the other hand, too much free Ca^2+^ could generate saline stress to the organism. This may partially explain the inhibitory effect of CaCl_2_ on the growth of the wild-type and mutant strains.

It has been proposed that the annexin peroxidase activity could protect the membranes against peroxidation (Jami et al., 2008) or locally terminate a peroxide-based signal within the membrane microdomains (Mortimer et al., 2009). Annexin involvement in such pathways is consistent with the exhibited peroxidase activity of its soluble and membrane-bound forms (Hofmann et al., 2000; Gorecka et al., 2007; Mortimer et al., 2008). The *ANXC7* protein seems to be different from the above ANXs with peroxidase activity, because *ΔANXC7* showed oxidative resistance to exogenous H_2_O_2_, and endogenous H_2_O_2_ levels within the *ΔANXC7* were lower than in the wild-type (**Figure 5**).

The peptide methionine sulfoxide reductase (Msr) can protect bacteria against oxidative damage from reactive nitrogen intermediates (St John et al., 2001). The cAMP-dependent PKA regulatory subunit (PKaR) is another stress-related protein that also involved in the response to oxidative stress and acted as a negative regulator of PKA (Norbeck and Blomberg, 2000); and the glycerol dehydrogenase 1 (Gcy1) activity is inversely correlated with the PKA activity in yeast (Norbeck and Blomberg, 2000). Therefore, Msr, PKaR, and Gcy1 are closely related to the resistance to oxidative stress. Actually, in *A. fumigatus*, the inactivation of PkaR increases the sensitivity of conidia to oxidative damage (Zhao et al., 2006); and the *A. fumigatus Msr* has been shown to be upregulated in response to reactive oxygen species (ROS) in an annexin C4 mutant (Khalaj et al., 2011). Both *ANXC7* and annexin C4 belong to the same C family of ANXs; thus, we hypothesized that the three genes are involved in the *ANXC7*-mediated response to oxidative stress, although their resistance to hydrogen peroxide is just the opposite (**Figure 5**). As expected, all the three genes were upregulated, and each was manifested to be upregulated at least 10-fold in *ΔANXC7* compared to the wild-type (**Supplementary Figure S4**).

Several members of the annexin family, which have long been known as PKC (Protein Kinase C) substrates, both *in vitro* and *in vivo* (Dubois et al., 1996; Rothhut, 1997), can promote membrane association of PKC isozymes (Ron and Mochly-Rosen, 1994). The PKC signaling pathways play crucial roles in maturation, hormone regulation, and other development related processes (Kwak et al., 1997); and although the specific reason is not clear, we speculate that the precocity and shedding of conidia may be related to the involvement of the *ANXC7* protein in the PKC pathway. Further experiments are still needed to determine the relevance of this hypothesis.

## Author Contributions

The experiments were conceived and designed by S-HZ and YW. The experiments were performed by HY and X-LX. The data was analyzed by X-LX, HY, L-NC, and YW. The paper was written by X-LX and S-HZ.

## Conflict of Interest Statement

The authors declare that the research was conducted in the absence of any commercial or financial relationships that could be construed as a potential conflict of interest.

## Footnotes

1 http://fungalgenomics.ca/wiki/Fungal_Genomes

2 http://dog.biocuckoo.org/

3 http://swissmodel.expasy.org/

4 https://wolfpsort.hgc.jp/
