# Supplementary material for: ANXC7 Is a Mitochondrion-Localized Annexin Involved in Controlling Conidium Development and Oxidative Resistance in the Thermophilic Fungus Thermomyces lanuginosus
Source: Front Microbiol. 2018 Sep 11;9:1770. doi: 10.3389/fmicb.2018.01770 (PMC6142879; doi:10.3389/fmicb.2018.01770)
Supplement: Supplementary file 1 [file Data_Sheet_1.docx]

**SUPPLEMENTARY MATERIAL**

Figure S1. Construction strategies of gene deletion and functional complementation vectors. (A) *ANXC7* gene deletion vector. LB: upstream flanking sequences of the *ANXC7* gene. RB: downstream flanking sequences of the *ANXC7* gene. *ANXC7*: the target gene. The genomic DNA of the 9W strain was used to amplify the upstream and downstream flanking sequences of *ANXC7*, which were cloned between the XhoI and EcoRI and the Xbal and HindIII sites, respectively, of the PXEH vector to generate the final replacement vector. HYG: hygromycin B phosphotransferase resistance gene. (B) The functional complementation vector. The coding sequence of *ANXC7* was amplified from 9W genomic cDNA and cloned between the Xbal and SalI sites of the pKD7-RED vector to generate the final complementation vector, pKD7-RED- ANXC 7. PH3: H3 promoter. DsRED2: red fluorescence marker. NEO: neomycin phosphotransferase 1I gene.

Figure S2. The *ANXC7* deletion strains were obtained and validated. (A) Colony morphology of the wild type, deletion mutant, and complementation strains grown on PDA plates at 50 °C for 5 days. (B) The target genes, hygromycin, left lateral genes, and hygromycin fragments were amplified with one of the genomic DNAs extracted from the above strains as a template. (C) The relative expression of the *ANXC7* gene in wild-type, deletion mutant, and complementation strains was detected via qPCR using cDNA as a template. The relative mRNA levels were calculated using the 2^-∆∆Ct^ method. The actin gene was used as an internal standard reference. The data represent means ± standard deviations (SD) of three experiments. Error bars represent standard deviation.

Figure S3. The H_2_O_2_ standard curve. 0–5 μM titanium–H_2_O_2_ complex was added into seven 15 ml centrifuge tubes, then a 300 r/min centrifugation for 10 min, and the supernatant was discarded. A total of 6 ml of 2M sulfuric acid was then added to each tube to dissolve the precipitate, and the absorbance of each tube was detected at 415 nm.

Figure S4. Relative expression of oxidative stress related genes. The relative mRNA levels were calculated using the 2^-∆∆Ct^ method. The actin gene was used as an internal standard reference. The data represent means ± standard deviations (SD) of three experiments. Error bars represent standard deviations. ** indicates a very significant difference at *P＜0.01*. *** indicates an extremely significant difference at *P＜0.001*.

**Table S1. Nucleotide sequences of primers used in this study**

| Primer name | Sequence |
| --- | --- |
| ANXC7-LB-F: | 5'-CCCTCGAGGCACTAACTCCGCATAACC-3’ |
| ANXC7-LB-R: | 5'-CGGAATTCGCAGGATTTCTGAAGACAAT-3’ |
| ANXC7-RB-F: | 5'-GCTCTAGACATAAATCCCTTTCAAGCA-3’ |
| ANXC7-RB-R : | 5'-CCCAAGCTTCACATCACCACGGACAAC-3’ |
| ANX-HYG-F ： | 5'-GCGACCGAGGAGGATGGGATA-3’ |
| ANX-HYG-R ： | 5'-TGCTCACCGCCTGGACGACT-3’ |
| ANXC7-F: | 5'-ATGGTTTGATTGCCAGGGTT-3’ |
| ANXC7-R: | 5'-CCTGACCGAAATGATGCTTGT-3’ |
| Canxc7-F: | 5'-GCTCTAGAGCATGTCGTCGAATCCTCCG-3’ |
| Canxc7-D: | 5'-GTCGACTTACGCGAGTAACGCCAC-3’ |
| RT-TlGcy1-F: | 5'-GATAGCAAGAAACACCTCCC -3’ |
| RT-TlGcy1-D: | 5'-CTCCAAATGCGTGATTCC -3’ |
| RT-TlMsr-F: | 5'-TCTACCGCAAGCACTTTG-3’ |
| RT-TlMsr-D: | 5'-TACGCTCCTGTTCCTCATC-3’ |
| RT-TlPkaR-F: | 5'-CAAAGTCATCACGCAAGG-3’ |
| RT-TlPkaR-D: | 5'-TGGTCCAGGGATACTACAGTC -3’ |
| Action-F： | 5'-TCATCACCGTTGACCCTTTC-3’ |
| Action-R： | 5'-ACGAGTCCTTCTGACCCATA-3’ |
| RT-ANX-F: | 5'-GCATGGAGGGCACCGTGAA -3’ |
| RT-ANX-D: | 5'-CTGGGTATGGGCCTGGTGG -3' |
| DsRED-P1 | 5'-AACCCGGGATGGCCTCCTCCGAGAACGTCATC-3' |
| DsRED-P2 | 5'-AATCTAGACAGGAACAGGTGGTGGCG-3' |
| NEO-SXF | 5'-GCACTAGTGAGGTCAACACATCAATGC-3' |
| NEO-SXR | 5'-TTTCTGAGTCAGAAGAACTCGTCAAGAAGGCG-3' |
