# Supplementary figures and images for: ANXC7 Is a Mitochondrion-Localized Annexin Involved in Controlling Conidium Development and Oxidative Resistance in the Thermophilic Fungus Thermomyces lanuginosus
Source: Front Microbiol. 2018 Sep 11;9:1770. doi: 10.3389/fmicb.2018.01770 (PMC6142879; doi:10.3389/fmicb.2018.01770)

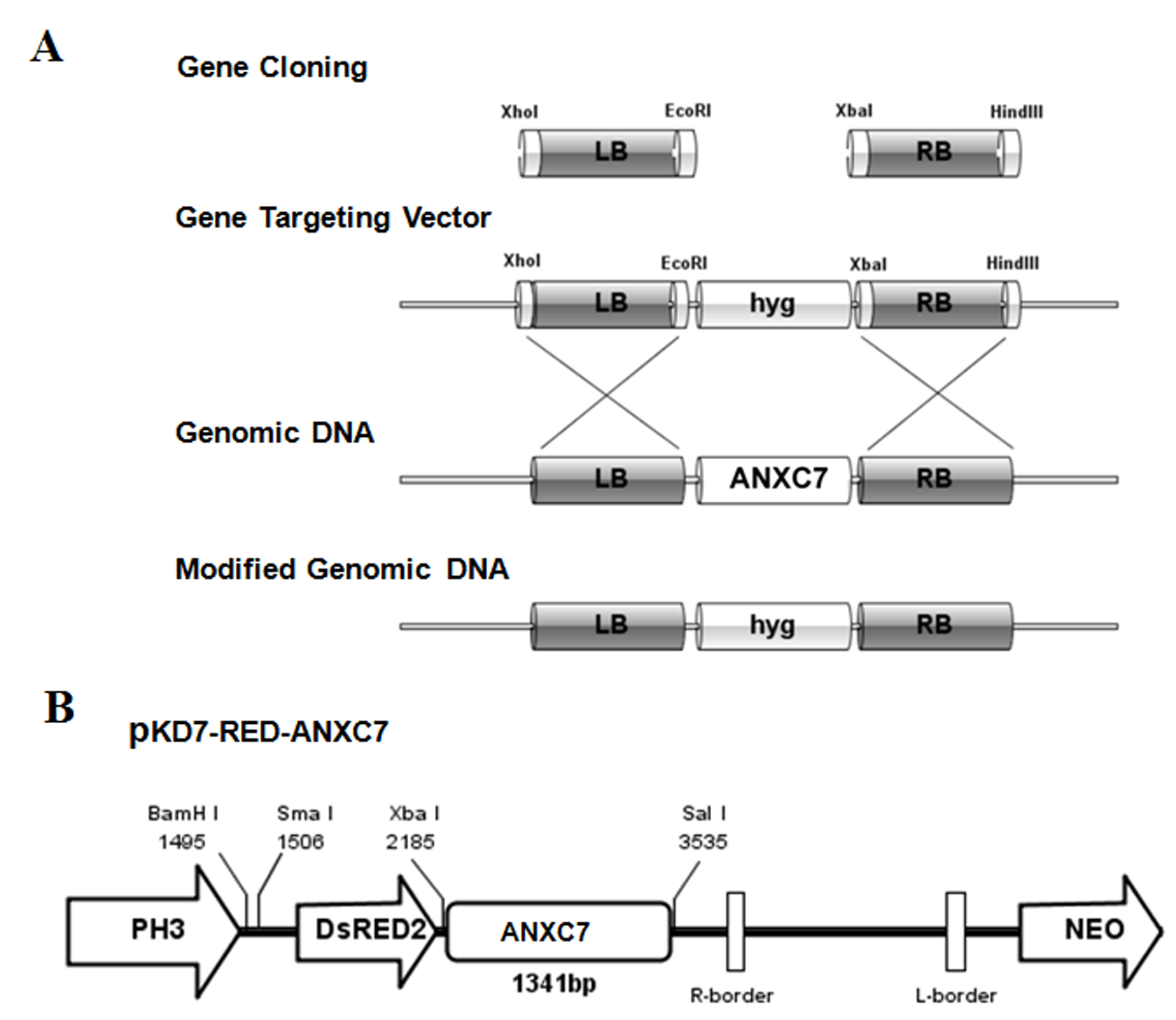

Supplement: Supplementary file 2 [file Image_1.TIF]

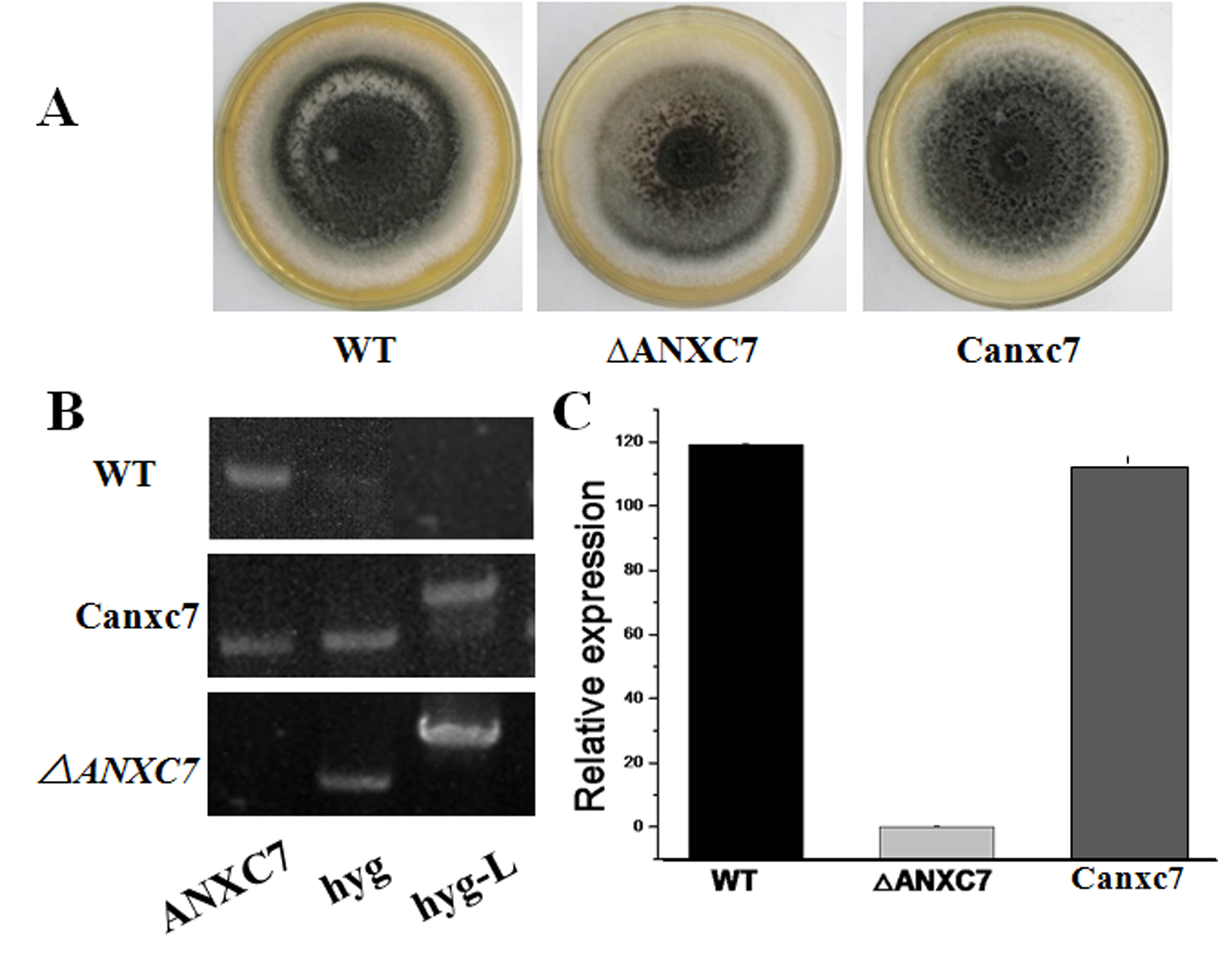

Supplement: Supplementary file 3 [file Image_2.TIF]

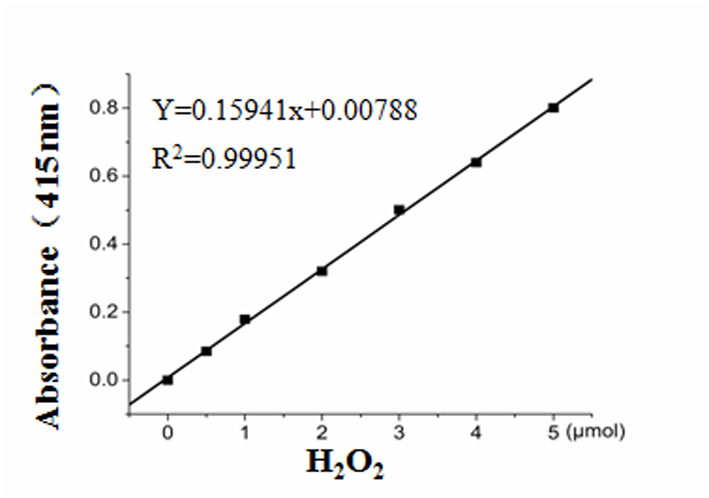

Supplement: Supplementary file 4 [file Image_3.TIF]

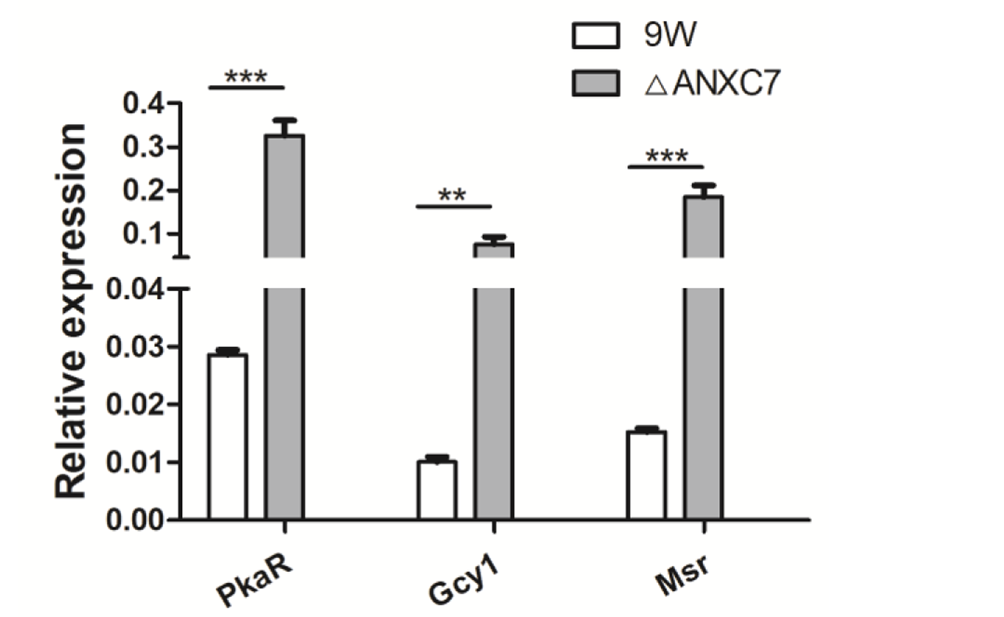

Supplement: Supplementary file 5 [file Image_4.TIF]
